# Factors associated with self-rated health status in university students: a cross-sectional study in three European countries

**DOI:** 10.1186/1471-2458-8-215

**Published:** 2008-06-18

**Authors:** Rafael T Mikolajczyk, Patrick Brzoska, Claudia Maier, Veronika Ottova, Sabine Meier, Urszula Dudziak, Snezhana Ilieva, Walid El Ansari

**Affiliations:** 1 Department of Public Health Medicine, School of Public Health, University of Bielefeld, Bielefeld, Germany; 2 Department of Epidemiology & International Public Health, School of Public Health, University of Bielefeld, Bielefeld, Germany; 3 Department of Prevention & Health Promotion, School of Public Health, University of Bielefeld, Bielefeld, Germany; 4 Institute of Family Sciences, Catholic University of Lublin, Lublin, Poland; 5 Department of Social, Work and Educational Psychology, Faculty of Philosophy, Sofia University "St. Kl. Ohridski", Sofia, Bulgaria; 6 Faculty of Sport, Health & Social Care, University of Gloucestershire, Gloucester, UK

## Abstract

**Background:**

Self-rated health status (SRHS) is a reliable and valid measure for assessing the subjective and objective health of individuals. Previous studies have either focused predominantly on the elderly or investigated only a narrow range of factors potentially associated with SRHS. In examining student populations, these past studies were limited to single countries. The objectives of this study were to assess which candidate variables were independently associated with SRHS in university students, to compare these variables by country and by gender, and to investigate which of the variables was most important as a rating frame for SRHS.

**Methods:**

The data is from the Cross-National Student Health Survey, conducted in 2005 in universities in Germany, Bulgaria, and Poland (n = 2103; mean age = 20.7 years). SRHS was assessed with a single question using a five-point scale ranging from "excellent" to "poor". The study also measured a wide range of variables including: physical and psychological health, studying, social contacts/social support, and socio-demographic status.

**Results:**

Psychosomatic complaints (considered an aspect of physical health and, adjusted for psychological health) were the most important indicators in forming a rating frame for students' SRHS. There were few differences in the effects of variables associated with SRHS by gender (well-being: a measure of psychological health) and the variables associated with SRHS by country (well-being and self-efficacy). The remaining variables showed homogenous effects for both genders and for all three countries.

**Conclusion:**

The results suggest that SRHS can be reasonably used to compare students' health across countries. SRHS is affected by different physical, psychological and psychosomatic aspects of health; however, its strongest association is with psychosomatic complaints.

## Background

Asking for a self-rating of health is a legitimate technique for assessing the health of individuals [1]. While self-rating of health is a good measure of objective and subjective health [2], it is also a feasible way to measure health in large-scale surveys [3,4]. Self-rating of health has been shown to have high reliability, validity and predictive power for a variety of illnesses and conditions [5]. Self-rated health has been extensively studied in older adult population groups, where a range of factors associated with self-rated health has been identified [6-8]. Much less is known about the self-rated health of younger populations. An exception is the international "Health Behaviour in School-Aged Children" study which contributed to the understanding of factors associated with self-rated health in school-aged children [9,10]. However, for young adults (e.g. university students) the available information remains limited in scope. On the one hand, university students have concerns, burdens and worries which are different from other population groups. On the other hand, these students face the dual stress of having academic challenges and achievements often within the face of financial limitations [11,12]. Hence unsurprisingly, in Sweden students were found to have lower perceived quality of life when compared with their working peers [13], and similar observations have been reported in the UK [14]. The published literature also suggests that young people preferentially employ psychological or behavioural factors as a rating frame for their health [3,15,16]. In contrast, for older people, physical well-being plays a more crucial role in assessing their health [3,8]. Given the observation that young adults differ from older people in their perception of health, a better understanding and a separate analysis of the factors associated with self-rated health status (SRHS) is needed for this younger age group. This is particularly true for university students, who represent an important and broad subpopulation of young adults.

Some studies of self-rated health exist for student populations in selected countries: e.g. USA [15], Canada [17], Hungary [16,18], and the UK [14]. Recent reports on the health of student populations in the USA have employed only crude analysis [19-21]. These studies cannot be directly compared with each other in terms of sample selection, measures and methods of analysis, thus they do not provide sufficient information regarding possible differences in self-rated health and the factors associated with it across countries. Conversely, some comparative cross-country studies of health in student populations do exist [22,23], however they have not assessed or reported findings in regard to the factors associated with SRHS. Thus there is a gap in the research concerning the SRHS in students across countries.

A recent review of inquiries which employed SRHS as an outcome variable found that such studies often omitted important variables or alternatively, did not provide adequate systematic analyses [24]. Most of these studies dealt with other age groups. Interestingly, the studies related to student populations were more complete in respect to the considered variables [14-18]. However, apart from lacking international comparisons, some issues remain unresolved. For example, the literature that addresses differences in SRHS for males and females has shown conflicting results: some studies found no significant differences between genders in bivariate analysis [18] or when controlling for other variables [15]. In contrast, other investigations have reported significant differences between genders in bivariate or stratified analyses. Yet these studies fell short of assessing the effect of gender using multivariable models [16,20,25,26]. Collectively, these concerns have formed the basis of the study described in this paper.

The present study had three specific aims: first, to investigate a wide range of variables potentially associated with SRHS in student populations, employing a cross-country comparison of students from three countries in Western (Germany), Central (Poland) and South Eastern (Bulgaria) Europe, second, to test whether these associations differed by gender and/or across participating countries, and third, to assess the comparative contributions of psychological, physical and other variables to the SRHS of student populations.

## Methods

### Study design and sample

The data analysed in this paper is from the Cross National Student Health Study (CNSHS). The CNSHS is a health survey that was conducted in 7 European countries between 1998 to 2005 [27]. This paper uses data collected in the survey conducted in 2005 at three universities; participants include 2103 first-year students at the University of Bielefeld, Germany; the Catholic University of Lublin, Poland; and Sofia University, Bulgaria. Ethical approvals for the study were obtained through the participating faculties. Participation in the survey was voluntary via a self-administered questionnaire distributed during lectures. The questionnaire employed was compiled and developed from different published sources, including validated instruments used in various populations [28-31], as well as questions developed specifically for this survey. The original version of the questionnaire was written in German and then translated into Bulgarian and Polish using two separate translations and expert consultation. Response rates were over 95% for both the Bulgarian and Polish samples, but varied from 60%–100% for the German sample depending on the surveyed groups, 85% on average (response rates were lower in large lecture rooms than in smaller seminars). Because each of the universities had unique academic structures composed of different faculties of various sizes, the surveys were implemented only in certain classes in an effort to achieve comparability between and representativeness for each of the participating universities. Collectively, the sample was comprised of students from 5 disciplines: natural sciences, humanities, social sciences, law, and economy, each contributing to about 20% of the sample.

### Measures

SPHS was assessed by the single item used in the 1998 German Federal Health Survey [32] (similar wording was also used by American College Health Association [20]). The item asked: "How would you rate your health in general?" over a 5-point scale response format (1 = "excellent", 2 = "very good", 3 = "good", 4 = "fair", 5 = "poor").

Table 1 outlines the five main areas believed to potentially influence students' health perceptions according to previous research. These included physical health/health related behaviours, psychological health/personality, variables related to studying, social contacts/social support, and socio-demographic characteristics of the participants.

**Table 1:** Areas that influence SRHS and variables used to measure them in this study

**Physical health/health related behaviours**	**Psychological health/Personality**	**Variables related to studying**	**Social contacts/Social support**	Socio-demographic characteristics*
> 2 visits to a doctor^+^	Sense of coherence	Importance of good grades (at university)	Having (intimate) relationship	Country
Physical activity	Self efficacy	Academic performance at the university (in comparison to peers)	Number of persons who could provide social support	Gender
BMI	Perceived stress	Burdens related to studying	Satisfaction with social support	Maternal education
Smoking status	Well being		Living location during term/semester	Sufficiency of income
Psychosomatic health complaints				

BMI: body mass index.

* Age was not included given little variation within the samples.

^+ ^As indicator of acute or chronic illness (see limitations).

Five variables were used to assess physical health/health related behaviours: having more than 2 visits to a doctor in the last six months (used as an indicator of severe acute or chronic illness); degree of physical activity (measured as frequency of physical activity over 20 minutes with increased pulse and respiration in a typical week, scored with a three-category response scale: less than once, 1–2 times, at least 3 times); Body Mass Index (BMI) based on self-reported weight and height and subdivided into three categories (< 20, 20–25, > 25 kg/m^2^); and smoking status (smoking in the last three months: daily, occasional, no). The fifth variable to assess physical health was a scale of psychosomatic health complaints assessed by a 22-item instrument measuring the frequency of occurrence of a range of complaints in the last 12 months (from 1 = "never" to 4 = "very often") [33]. This scale covered many complaints: stomach trouble/heartburn; back pain; fatigue; breathing difficulties; trembling hands; rapid heart beat/circulatory problems/dizziness; diarrhoea; constipation; headaches; sleep disorder/insomnia; nightmares; difficulties concentrating; neck and shoulder pain; abdominal problems; mood swings; trembling; depressive mood; speech impediment; weight gain/weight loss; lack of appetite; nervousness/anxiety; and fear/phobia. Since the internal reliability for this scale was high (Cronbach's alpha = 0.88), a single score (mean over all items) of the psychosomatic health complaints for each person was used in the subsequent analysis.

Psychological health/personality were collectively assessed using four validated scales: Sense of coherence (mean of 7 items, 7-point scale from "low" to "high") [28]; self-efficacy (mean of 10 items, 4-point scale from "low" to "high") [30]; perceived stress (mean of 14 items, 5-point scale from "little" to "much") [29]; and the WHO-Five Well-being Index (WHO-5) (sum of 5 items, 5-point scale "low" to "high") [31].

The variables related to studying at a university were two single items: the importance of achieving good grades at the university (5-point scale from "very important" to "not important at all"); and academic performance in the university in comparison to one's peers (5 point scale from "much better" to "much worse"). In addition, one more scale was developed for the purpose of this inquiry: burdens related to studying (9 items: how much do you feel burdened by: studying in general, exams and assignments, lack of practical relevance of coursework, anonymity in university, poor career prospects, problems with peers, course work load, isolation at the university, and lack of time for studying, all scored from 1 = "not at all" to 6 = "very much"). The items were highly correlated with Cronbach's alpha = 0.81; therefore we used solely the mean of all items in the analysis.

The area of social contacts/social support was assessed with two items regarding existing relationships (having an intimate partnership and living with parents during term/semester). Another two items assessed social support, namely the number of persons who could provide social support (four categories: none, 1, 2–3 and more than 3 persons), as well as the satisfaction with this support (1 = "very satisfied" to 5 = "not satisfied at all").

Finally, five variables were used to assess participants' socio-demographic characteristics: country of the survey, gender, maternal education (maternal education was used which provided more variability than paternal education within the samples) and subjective sufficiency of income (1 = "totally sufficient" to 4 = "not sufficient at all"). We did not analyse age dependency of SRHS as the age distribution in the samples did not overlap well between countries and provided little variation within countries.

### Statistical analysis

Descriptive statistics were performed using tabulations by gender and country. Mann-Whitney-U-test and Pearson's chi-square test were used to test for differences across countries or between genders. Bivariate associations with SRHS were tested by Mann-Whitney-U- and Kruskal-Wallis-H-test for categorical and by Kendall-tau-b Spearman rank correlations for continuous variables. (We treated scales as continuous variables.) Because the distribution of SRHS (apart from being discrete) resembled the shape of a normal distribution, a General Linear Model was used for the analysis of independent associations in the multivariable model. Initially, all variables significantly associated with SRHS in the bivariate analysis were included in the main effects model. This model was then reduced in a stepwise process using Wald test criterion. Since potential effect modifications by gender were expected, gender was contained in the model, although its main effect was not significant. In the next step all two-way interactions for gender and country with any other variables which remained significant were included in the model. Again the insignificant interaction terms were removed in a stepwise manner. In order to compare the effect sizes of variables in the five different areas under investigation for associations with SRHS (see above), partial Eta-square statistic was used as it allows effect sizes to be compared across different variables, independent of any other variables included in the model [34,35]. The analysis was performed with SPSS^® ^for Windows version 12. For all tests, significance level was set at p < 0.05.

## Results

### Description of the sample

This sample of university students was predominately female (65.1%) with the highest proportion being in Poland (71.3%) (Table 2).Overall, male students were slightly older (mean age 21.1 years vs. 20.6 years in females). While females reported having an intimate partner more often than their male counterparts (49.1% vs. 38.3%), male students were more likely to live in their parents' home during terms/semesters (44% vs. 31.7% in females). Males and females did not differ in respect to maternal education; however, pronounced differences existed across countries with Bulgaria having a much larger proportion of mothers with higher education (high school degree or more) than in the two other countries. Students differed in weight categories (underweight = BMI < 20, normal weight = BMI 20–25, overweight = BMI > 25) by gender and country, with a higher prevalence of underweight in women and in Bulgaria. Students did not differ in regard to smoking by gender, but smoking was less prevalent in Poland than in the other countries. About 8.7% of the surveyed students rated their health as excellent, 35.8% reported it as very good, 45.6% as good, 8.9% as fair, and 1% felt that their health was poor.

**Table 2:** Selected characteristics of the sample by gender and country

	**Whole Sample**	**Gender**^a^			**Country**			
**Variable**		**Female**	**Male**	p-value*	**Germany**	**Poland**	**Bulgaria**	p-value*

	N = 2103	N = 1331	n = 712		n = 803	n = 591	n = 709	
Female [%]	65.1				57.7	71.3	68.3	< 0.001
Age [Mean (SD)] in years	20.7 (3.1)	20.6 (2.9)	21.1 (3.3)	< 0.001	22.1 (3.2)	20.2 (3.2)	19.7 (2.0)	< 0.001
Having a boy-/girlfriend [%]	45.4	49.1	38.3	< 0.001	55.6	35.5	42.3	< 0.001
Living with parents during semester [%]	36.0	31.7	44.0	< 0.001	35.6	29.8	41.2	< 0.001
Maternal higher education (high school or better) [%]	37.6	36.6	39.4	NS	24.1	26.0	61.8	< 0.001
BMI (kg/m^2^) [%]				< 0.001				< 0.001
< 20	32.9	43.6	13.0		20.4	34.6	44.5	
20 to < 25	55.5	48.8	68.0		61.8	56.2	48.6	
≥ 25	11.6	7.6	19.0		17.7	9.2	6.9	
Smoking status [%]				NS				< 0.001
Daily	19.3	19.7	18.9		22.3	10.4	23.3	
Occasionally	15.3	15.4	15.3		15.4	13.6	16.6	
Non-smoker	65.3	64.9	65.8		62.3	76.0	60.1	

^a^: information about gender was missing for 60 (3%) participants; NS= not significant;

*: Pearson's chi-square or Mann-Whitney-U tests for comparison between genders; and for comparison between countries

### Associations between the variables under investigation and SRHS in bivariate analysis

Table 3 demonstrates that all of the investigated variables with dichotomous/categorical response scales showed associations with SRHS [except for BMI and having an (intimate) relationship to a boy- or girlfriend which were not associated to SRHS]. For instance, male students rated their health better than females. There were also differences by country, with the best health rating in Bulgaria, followed by Germany then Poland. Students who reported more than two visits to a doctor in the last six months rated their health lower than those who reported fewer or no visits, and regular smokers rated their health lower than non- or occasional smokers. Among the variables related to social contacts/social support, having a boy- or girlfriend was not associated with health, but living during terms/semesters with parents was associated with better health. Similarly, Table 4 shows that all of the continuous variables under investigation (apart from the importance of good grades) were significantly associated with SRHS in the bivariate analysis. The correlation coefficients varied between 0.1 and 0.3, with the strongest correlation occurring between psychosomatic complaints and self-rated health.

**Table 3:** Bivariate analysis: associations between SRHS and variables scored on dichotomous/categorical response scales

**Variable**	**SRHS Mean (SD)**
Gender***	
Female	2.67 (0.77)
Male	2.40 (0.85)
Country***	
Germany	2.62 (0.77)
Poland	2.78 (0.79)
Bulgaria	2.36 (0.82)
BMI	
< 20	2.59 (0.81)
20 to < 25	2.55 (0.81)
>= 25	2.65 (0.86)
> 2 visits to a doctors in the last six months***	
No	2.46 (0.79)
Yes	2.75 (0.81)
Smoking status (in the last 3 months)*	
Daily	2.66 (0.81)
Occasionally	2.54 (0.86)
Non-smoker	2.56 (0.80)
Living location during term**	
Together with parents	2.49 (0.82)
Alone, with room mates/partner, other	2.63 (0.80)
Having (intimate) relationship	
Yes	2.56 (0.79)
No	2.58 (0.82)
Maternal education	
No formal education	3.13 (0.74)
Grades 1–8	2.81 (0.73)
Grades 9–11	2.58 (0.73)
High school degree	2.64 (0.81)
Bachelor/Master/Ph.D. or equivalent	2.46 (0.86)

*p < 0.05; **p < 0.01; ***p < 0.001 in Mann-Whitney-U tests and Kruskal-Walis-H test

**Table 4:** Bivariate analysis: associations between SRHS^a ^and variables with continuous response scales

**Variable**	**Correlations (Kendall tau-b)**
Psychosomatic health complaints^b^	0.317*
Physical activity^c^	0.146*
Sence of Coherence^d^	0.273*
Perceived stress^e^	0.268*
Self-efficacy^f^	-0.245*
Well-being^g^	-0.270*
Sufficiency of income^h^	0.089*
Importance of good grades^i^	0.034
Academic performance at the university^j^	0.161*
Burdens related to studying^k^	0.196*
Satisfaction with social support^a^	0.121*
Number of persons could provide social support^l^	-0.043*

*p < 0.05; **p < 0.01; ***p < 0.001; ^a^1 = "excellent" to 5 = "poor"; ^b^1 = "never" to 4 = "very often"; ^c^1 = "less then once per week", 2 = "1 or 2 times", and 3 = "at least 3 times"; ^d^1 = "very low" to 7 = "very high"; ^e^1 = "little stress" to 5 = "much stress"; ^f^1 = "low" to 4 = "high"; ^g^0 = "lowest possible well-being" to 25 = "highest possible well-being"; ^h^1 = "totally sufficient" to 4 = "not sufficient at all";^i^1 = "very important" to 4 = "not at all important"; ^j^1 = "much better (than peers)" to 6 = "much worse (than peers)"; ^k^1 = "low" to 6 = "high"; ^l^1 = "no person" to 4 = "more than 3 persons";

### Variables associated independently with SRHS in multivariable analysis

Before assessing the interaction effects, 8 variables displayed significant associations with SRHS in the multivariable analysis. Gender was contained in the model for further analysis despite not displaying a significant association with SRHS and all interactions with gender and country were investigated. The final model included 9 main effects, along with 2 interactions with country and 1 with gender (Table 5). No interaction with either country or gender was found for sufficiency of income, psychosomatic complaints, acute or chronic illness (assessed by the indicator variable of > 2 visits to a doctor), physical activity and sense of coherence. Higher psychosomatic complaints score and > 2 visits to a doctor in last six months were both associated with poorer health. The same was true for less sufficient income, but the effect of income was much weaker. Conversely, higher physical activity and a better sense of coherence were both associated with better health.

**Table 5:** Multivariable analysis: variables independently associated with SRHS

**Variables**	**β**	**SE**	**95%-CI**	**Partial Eta-square**
Gender				0.002
Male	Ref			
Female	-0.20	0.11	(-0.41, 0.02)	
Country				0.001
Bulgaria	Ref			
Germany	-0.29	0.27	(-0.82, 0.24)	
Poland	-0.23	0.27	(-0.75, 0.30)	
Sufficiency of income^a^	0.05*	0.02	(0.01, 0.10)	0.004
Psychosomatic complaints^b^	0.44***	0.06	(0.33, 0.55)	0.043
> 2 visits to a doctors in the last six months				0.028
Yes	Ref			
No	-0.27***	0.04	(-0.36, -0.19)	
Physical activity^c^	-0.09**	0.03	(-0.14, -0.03)	0.007
Well-being^d^	-0.03***	0.01	(-0.05, -0.02)	0.011
Self-efficacy^e^	-0.27***	0.08	(-0.42, -0.12)	0.009
Sense of coherence^f^	-0.06*	0.03	(-0.12, -0.01)	0.004
Well-being by				0.006
Country (Bulgaria)	Ref			
Country (Germany)	-0.02*	0.01	(-0.04, -0.002)	
Country (Poland)	0.01	0.01	(-0.01, 0.03)	
Well-being by				0.004
Gender (Male)	Ref			
Gender (Female)	0.02*	0.01	(0.003, 0.03)	
Self-efficacy by				0.007
Country (Bulgaria)	Ref			
Country (Germany)	0.30**	0.10	(0.11, 0.50)	
Country (Poland)	0.20	0.11	(-0.01, 0.41)	

^†^Positive coefficients β indicate decrease in health on the 5 point scale; in contrary negative coefficients indicate a better SRHS; Ref: reference category; ^a ^per unit of the 4 point scale from 1 = "totally sufficient" to 4 = "not sufficient at all"; ^b^1 = "never" to 4 = "very often"; ^c^1 = "less then once" to 3 = "at least 3 times"; ^d^0 = "lowest possible well-being" to 25 = "highest possible well-being"; ^e^1 = "low" to 5 = "high"; ^f^1 = "very low" to 7 = "very high"; Significance of Wald test for the coefficient = 0: *p < 0.05, **p < 0.01, ***p < 0.001

**Figure 1:**
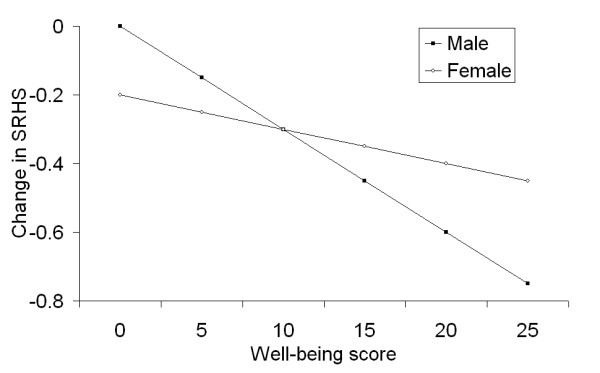
**Combined effects of well-being and gender on SRHS**. Note: y-axis indicates the difference in SRHS at each well-being score for both genders, or the changes in SRHS score when different well-being scores are considered.

**Figure 2:**
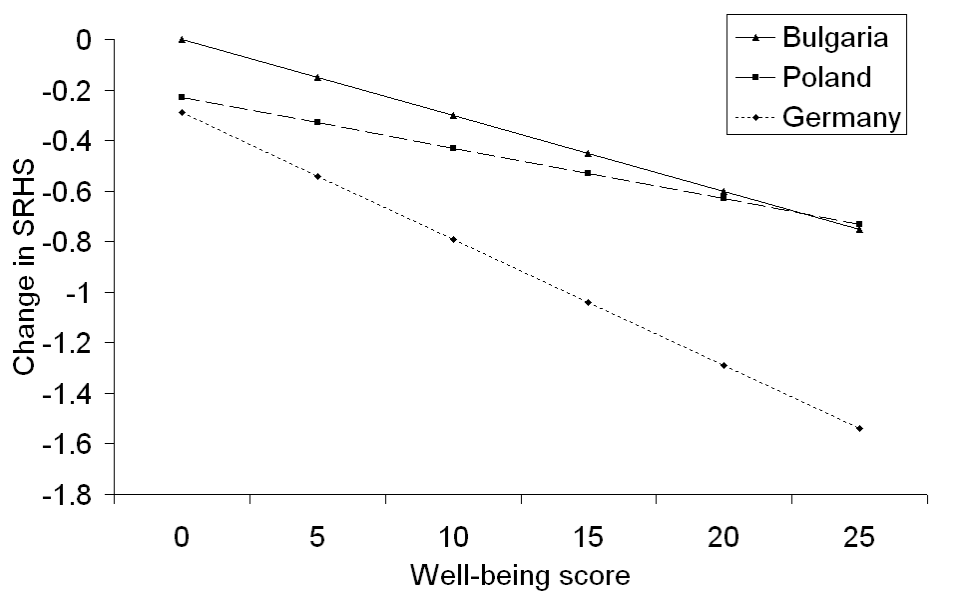
**Combined effects of well-being and country on SRHS**. Note: y-axis indicates the difference in SRHS at each well-being score between the countries, or the changes in SRHS score when different well-being scores are considered.

**Figure 3:**
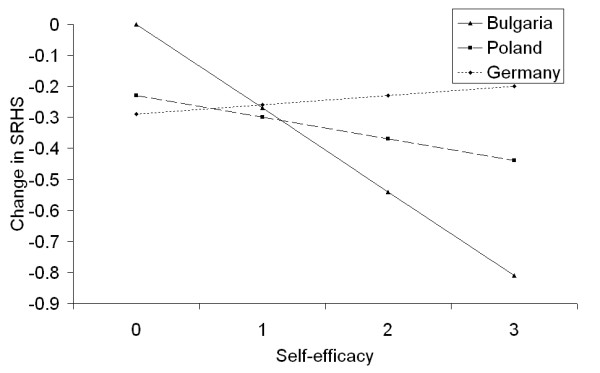
**Combined effects of self-efficacy and country on SRHS**. Note: y-axis indicates the difference in SRHS at each self-efficacy score between the countries, or the changes in SRHS score when different self-efficacy scores are considered.

The main effect of gender indicated a better level of health in male students, but due to the presence of interactions with other variables, the final interpretation had to consider these interactions. Thus, females who had low well-being scores reported better SRHS than males with low well-being scores (a well-being score below 10 was considered low- the mean well-being score in this sample was 12.5). Conversely, males with higher well-being scores reported better SRHS than females with higher scores (Figure 1). There was also an interaction between well-being and country, with similar SRHS values in Poland and Germany for individuals with low well-being scores, and a large difference between Germany and the two Slavic countries (Figure 2). Both figures also indicated that the effect of well-being was greater in female students and in Germany than in the remaining countries. In contrast, the effect of self-efficacy was much greater in Bulgaria than in Poland or Germany (Figure 3). The sample's mean self-efficacy score was 2.8 (2.9 for both Germany and Bulgaria, 2.6 for Poland), and at this self-efficacy score, Bulgarian students had the highest SRHS of all three countries. Finally, none of the variables related to studying or social contacts were independently associated with SRHS.

### Importance of the five different areas as rating frame for SRHS in students

The partial Eta-square indicated that psychosomatic complaints had the strongest effect on SRHS (Table 5, last column). However, psychological aspects as measured by well-being or self-efficacy were also important. None of the physical health measures interacted with gender or country, whereas two of the psychological measures displayed different effects with either gender or country. Socio-demographic variables were significantly associated with SRHS, although their contributions to individual SRHS were negligible. Again, variables related to areas of study at the university or social contacts were not associated with SRHS after controlling for the other variables. Thus neither an area of study nor measures of social contact contributed to a framework for students' rating of their health.

## Discussion

Attention on the health of university students has increased in recent years [23,36,37]. Acquiring and compiling knowledge about these population groups is imperative in creating health promotion activities to meet the needs and concerns of university students [14]. Designing appropriate interventions for these groups requires insight into and an understanding of the various factors associated with students' health. The present study examined a variety of factors associated with SRHS among university students from three European countries.

In line with previous inquiries, many students in this study rated their health as excellent or very good (e.g. [19-21,23]). From the initial five areas under investigation, only three proved to be independently associated with SRHS in students. Whereas physical, psychological aspects and socio-demographic characteristics displayed independent associations with SRHS, the area of social contacts and variables related to studying did not. Several of the variables (e.g. sufficiency of income, psychosomatic complaints, physical activity and sense of coherence) exhibited similar effects for both genders and in all three countries. In contrast, the effect of well-being was modified by gender and country, while the influence of self-efficacy was modified only by country. The effect of gender did not differ by country. We did not assess the three-way interaction between gender, country and well-being.

Some previous studies have postulated a gender difference in relation to SRHS, others have not. The observation that gender was not significantly associated with SRHS prior to considering the interactions might shed new light on these apparently contradictory findings. Although health perceptions are likely to be gender-specific (since gender is associated with many other health outcomes) [38], gender differences in variables associated with SRHS usually occur only for self-rated psychological health: with males rating this type of self-rated health higher [17]. This is consistent with our findings. Such findings also lend further support to Mantzavinis et al.'s [24] recommendations that analyses of SRHS should consider interactions among the variables investigated.

Steptoe and Wardle [22] have suggested that there are culturally diverging concepts and levels of valuation of health between Eastern and Western European students, with Western European presenting more favourable ratings. In our analysis the difference as suggested by Steptoe and Wardle was only found to be true among students reporting high well-being scores while their self-efficacy was relatively low. This finding is consistent with the hypothesis investigated by Steptoe and Wardle that the cause of differences in health perception between Eastern and Western Europe might be due to a lower perception of control in Eastern European countries due to instabilities during political and economic transitions. Steptoe and Wardle's analysis was based on data collected around 1990, a time when perception of control might have systematically differed between Eastern and Western European countries. In our study, a similar average self-efficacy score was found for students from all three countries, probably a consequence of improvements of the situation in Eastern Europe since the time of economic and political transition. This could help explain the relatively small differences in self-efficacy and SRHS in the countries examined in this paper.

Psychosomatic complaints and having > 2 visits to a doctor in the last six months displayed the strongest association with SRHS in our analysis, indicating the importance of physical health in the perception of general health for university students. A previous study using open-ended questions following a self-rating of health found that adolescents and young adults more often referred to their positive health behaviours in justifying their rating than older persons who were more likely to refer to physical health problems [3]. A qualitative study in middle-aged population indicated that health is primarily understood as absence of ill health, modified by disease specific aspects like duration, severity and consequences for everyday life [39]. Two further studies [15,16], using cross-sectional samples like our study, found that well-being (or psychological health) was the most important aspect for university students. While young people are less likely to suffer from chronic diseases than older people, for young people with chronic diseases still this aspect might be important for their rating of health. Also using a more common condition related to physical health like psychosomatic complaints might result in higher importance of physical health for SRHS. The prevalence of psychosomatic complaints increases steadily and considerably during the school age and complaints are highly prevalent in older school-aged children [40,41]. Nevertheless, Piko [16] found that well-being explained a higher fraction of variance in SRHS than psychosomatic complaints in her sample. Her assessment is based on a forward stepwise regression model, while we evaluated partial Eta square estimates from a mutually adjusted model, which is more correct for models assessing association rather than prediction. Also, we used a different measure of well-being in our study (while the assessment of psychosomatic complaints was very similar). It is not clear whether the difference in findings can be explained by the statistical methods used or by the different well-being measure. However, the issue is further complicated by the classification of psychosomatic complaints; i.e. it is worth discussing whether such complaints truly belong to physical or to psychological health. One previous study even used a complaints score similar to the one employed in this study as a general measure of self-rated health [26], which further complicates the interpretation. Since gender dependency in respect to self-rated health has been postulated for psychological rather than for physical aspects [17], the lack of interaction between gender and psychosomatic complaints suggested the stronger connection of the latter – psychosomatic complaints- to physical health. Additionally, in our model, the effects of psychological factors were controlled for in the association between psychosomatic complaints and SRHS. Thus, the effect of the psychosomatic complaints obtained in the final model is the 'net' effect which is not mediated by any psychological factors. In addition, the WHO-5 scale applied in our analysis draws its name from the general well-being item but can also be used for the assessment of depression [42]. Thus, by including this scale in the model, we were able to simultaneously control for the effects of different perceptions of health caused by depressive symptoms, which are frequent among university students [43-45].

This study has strengths: the relatively large sample size and the use of the same study design and questionnaire in three European countries with different socio-demographic profiles and cultural backgrounds. The analysis covered a wide range of factors, employing several variables for each of the areas under investigation; while differences in variables related to SRHS for gender and country were formally tested using interactions. Since SRHS is a useful indicator in health sciences, this inquiry has contributed to a reversal of two trends in the published literature: studies either employing too few variables, ultimately resulting in a constricted view and scope of self-rated perceptions of health, or alternatively, focusing predominantly on elderly populations. Hence, this study has bridged these gaps and contributed to the literature focusing on a young adult population and analysing a broad range of factors associated with SRHS. Both of these critical aspects have been highlighted in literature as being insufficiently considered [16,24].

The study has also some limitations. Findings are based on self-reported data with no validation undertaken. SRHS is genuinely a subjective measure of one's health, but the number of visits to a doctor could have been externally validated. Furthermore, cross-sectional approaches allow conclusions about associations, not causations. It could be that observed associations are reinforced by reversed causality, whereby not only a given behaviour or condition leads to a decreased SRHS, but also a decreased SRHS influences the behaviour/condition. Since this inquiry examined only one university per country, differences between countries could be in fact differences between universities. The questionnaire did not contain a direct question assessing the presence of severe, acute, or chronic illness. Instead, we used the number of visits to a medical doctor as a proxy for having such illnesses (> 2 visits in last 6 months). This could have had two effects: first, some students could have consulted a doctor more often without having a severe, acute, or chronic illness; conversely, some of chronically ill patients may not see a doctor as frequently (e.g. when on stable medication). Also, some students may have repeatedly visited a doctor because of mental health conditions. However, despite some potential misclassification, students experiencing illness are more likely to be found in the group visiting doctors more frequently and vice versa. For the statistical analysis we used the framework of a general linear model despite the outcome variable having only five discrete values, implicitly assuming that the differences between subsequent values of the scale were equal and that the normal residual error was well approximated.

## Conclusion

The present study examined factors associated with SRHS in a population of young adults who were currently university students in three European countries. Psychosomatic complaints exhibited the strongest effect as a rating frame for SRHS. While most of the variables associated with SRHS showed homogenous effects for all three countries, for two variables, a significant interaction with country existed: well-being and self-efficacy. It seems that SRHS can reasonably be used in studies of young adults in different countries; however, the two potential effect modifiers should be assessed. In the future, SRHS would benefit from further investigations using populations with greater diversity in European and other countries.

## Competing interests

The authors declare that they have no competing interests.

## Authors' contributions

RTM developed the questionnaire and study design, supervised the analysis and contributed to the final version of the manuscript; PB, CM, VO developed the research question, performed initial analysis and drafted the first version of the manuscript; SM, UD and SI participated in the development of the questionnaire; performed data collection and made comments on the manuscript; WEA participated in providing comments in the data analysis, participated in writing the final version of the manuscript and in the subsequent revisions. All authors read and approved the final manuscript.

## Pre-publication history

The pre-publication history for this paper can be accessed here:



## References

[B1] Idler EL, Benyamini Y (1997). Self-rated health and mortality: a review of twenty-seven community studies. J Health Soc Behav.

[B2] Maddox GL, Douglass EB (1973). Self-assessment of health: a longitudinal study of elderly subjects. J Health Soc Behav.

[B3] Krause NM, Jay GM (1994). What do global self-rated health items measure?. Med Care.

[B4] Mechanic D, Hansell S (1987). Adolescent competence, psychological well-being, and self-assessed physical health. J Health Soc Behav.

[B5] Lundberg O, Manderbacka K (1996). Assessing reliability of a measure of self-rated health. Scand J Soc Med.

[B6] Fylkesnes K, Forde OH (1992). Determinants and dimensions involved in self-evaluation of health. Soc Sci Med.

[B7] Shields M, Shooshtari S (2001). Determinants of self-perceived health. Health Rep.

[B8] Johnson RJ, Wolinsky FD (1993). The structure of health status among older adults: disease, disability, functional limitation, and perceived health. J Health Soc Behav.

[B9] Torsheim T, Currie C, Boyce W, Samdal O (2006). Country material distribution and adolescents' perceived health: multilevel study of adolescents in 27 countries. J Epidemiol Community Health.

[B10] Torsheim T, Ravens-Sieberer U, Hetland J, Valimaa R, Danielson M, Overpeck M (2006). Cross-national variation of gender differences in adolescent subjective health in Europe and North America. Soc Sci Med.

[B11] Kouzma NM, Kennedy GA (2004). Self-reported sources of stress in senior high school students. Psychol Rep.

[B12] Powers CB, Wisocki PA, Whitbourne SK (1992). Age differences and correlates of worrying in young and elderly adults. Gerontologist.

[B13] Vaez M, Kristenson M, Laflamme L (2003). Perceived quality of life and self-rated health among first-year university students. A comparison with their working peers.. Soc Indic Res.

[B14] Stewart-Brown S, Evans J, Patterson J, Petersen S, Doll H, Balding J, Regis D (2000). The health of students in institutes of higher education: an important and neglected public health problem?. J Public Health Med.

[B15] Garrity TF, Somes GW, Marx MB (1978). Factors influencing self-assessment of health. Soc Sci Med.

[B16] Piko B (2000). Health-related predictors of self-perceived health in a student population: the importance of physical activity. J Community Health.

[B17] Vingilis E, Wade TJ, Adlaf E (1998). What factors predict student self-rated physical health?. J Adolesc.

[B18] Piko B, Barabas K, Boda K (1997). Frequency of common psychosomatic symptoms and its influence on self-perceived health in a Hungarian student population. Eur J Public Health.

[B19] UTPA UTPA Health Education Online Survey Results. http://www.oire.utpa.edu/publications/HealthEducationSurvey_Jan06.pdf.

[B20] ACHA National College Health Assessment. Reference Group Data Report 2005.. http://www.acha.org/projects_programs/NCHA_docs/ACHA-NCHA_Reference_Group_Report_Fall2005.pdf.

[B21] USC USC American College Health Association National College Health Assessment Report 2005. http://www.usc.edu/student-affairs/Health_Center/docs/ncha.2005.pdf.

[B22] Steptoe A, Wardle J (2001). Health behaviour, risk awareness and emotional well-being in students from Eastern Europe and Western Europe. Soc Sci Med.

[B23] Stock C, Kücük N, Miseviciene I, Guillen-Grima F, Petkeviciene J, Aguinaga-Ontoso I, Kramer A (2003). Differences in health complaints among university students from three European countries. Prev Med.

[B24] Mantzavinis GD, Pappas N, Dimoliatis ID, Ioannidis JP (2005). Multivariate models of self-reported health often neglected essential candidate determinants and methodological issues. J Clin Epidemiol.

[B25] Vaez M, Laflamme L (2003). Health behaviors, self-rated health, and quality of life: a study among first-year Swedish university students. J Am Coll Health.

[B26] von Bothmer MI, Fridlund B (2003). Self-rated health among university students in relation to sense of coherence and other personality traits. Scand J Caring Sci.

[B27] El Ansari W, Maxwell AE, Mikolajczyk RT, Stock C, Naydenova V, Kraemer A (2007). Promoting Public Health: Benefits and Challenges of a Europeanwide Research Consortium on Student Health. Cent Eur J Public Health.

[B28] Antonovsky A (1993). The structure and properties of the sense of coherence scale. Soc Sci Med.

[B29] Cohen S, Kamarck T, Mermelstein R (1983). A global measure of perceived stress. J Health Soc Behav.

[B30] Schwarzer R, Jerusalem M, Weinman J, Wright S, Johnston M (1993). Generalized Self-Efficacy Scale. Measures in health psychology: A user’s portfolio Causal and control beliefs (35-37).

[B31] WHO (1998). World Health Organization info package: Mastering depression in primary care..

[B32] Potthoff P, Schroeder E, Reis U, Klamert A (1999). [Process and results of field work concerning the Federal Health Survey]. Gesundheitswesen.

[B33] Hurrelmann K, Kolip P (1994). [Der Jugendgesundheitssurvey].. Presseinformationsdienst des SFB 227, No 11.

[B34] Breaugh JA (2003). Effect size estimation: Factors to consider and mistakes to avoid.. Journal of Management.

[B35] Pierce CA, Block RA, Aguinis H (2004). Cautionary note on reporting eta-squared values from multifactor anova design. Educational and Psychological Measurement.

[B36] Kramer A, Prufer-Kramer L, Stock C, Tshiananga JT (2004). Differences in health determinants between international and domestic students at a German university. J Am Coll Health.

[B37] Stock C, Kücük N, Miseviciene I, Petkeviciene J, Krämer A (2004). Misperceptions of body weight among university students from Germany and Lithuania. Health Education.

[B38] Anson O, Paran E, Neumann L, Chernichovsky D (1993). Gender differences in health perceptions and their predictors. Soc Sci Med.

[B39] Manderbacka K (1998). Examining what self-rated health question is understood to mean by respondents. Scand J Soc Med.

[B40] Haugland S, Wold B, Stevenson J, Aaroe LE, Woynarowska B (2001). Subjective health complaints in adolescence. A cross-national comparison of prevalence and dimensionality. Eur J Public Health.

[B41] Sundblad GM, Saartok T, Engstrom LM (2006). Prevalence and co-occurrence of self-rated pain and perceived health in school-children: Age and gender differences. Eur J Pain.

[B42] Lowe B, Grafe K, Zipfel S, Witte S, Loerch B, Herzog W (2004). Diagnosing ICD-10 depressive episodes: superior criterion validity of the Patient Health Questionnaire. Psychother Psychosom.

[B43] Wardle J, Steptoe A, Gulis G, Sartory G, Sek H, Todorova I, Vogele C, Ziarko M (2004). Depression, perceived control, and life satisfaction in university students from Central-Eastern and Western Europe. Int J Behav Med.

[B44] Dyrbye LN, Thomas MR, Shanafelt TD (2006). Systematic review of depression, anxiety, and other indicators of psychological distress among U.S. and Canadian medical students. Acad Med.

[B45] Bostanci M, Ozdel O, Oguzhanoglu NK, Ozdel L, Ergin A, Ergin N, Atesci F, Karadag F (2005). Depressive symptomatology among university students in Denizli, Turkey: prevalence and sociodemographic correlates. Croat Med J.

